# Skills Training of Health Workers in the Use of a Non Surgical Device (PrePex) for Adult Safe Male Circumcision

**DOI:** 10.1371/journal.pone.0104893

**Published:** 2014-08-13

**Authors:** Moses Galukande, Kevin Duffy, Jean Paul Bitega, Nick Wooding

**Affiliations:** 1 International Hospital Kampala, and Surgery Department, Mulago Hospital, Kampala, Uganda; 2 International Medical Group, Kampala, Uganda; 3 Kanombe Hospital, Kigali, Rwanda; 4 International Health Sciences University, Kampala, Uganda; Centers for Disease Control and Prevention, United States of America

## Abstract

**Background:**

Safe Male Circumcision (SMC) is a proven approach for partial protection of men from acquisition of HIV infection. Several sub-Saharan African countries have a target to circumcise 80% of males aged 15 to 49. The use of devices such as PrePex would aid scaling up of SMC. Since most health workers would have no prior experience with use of devices, skills training is needed. This paper explores a skills transfer model at an urban site in Uganda.

**Objective:**

To assess the practicability and feasibility of rapid short duration training for safe PrePex device use.

**Methods:**

A prospective study, conducted over 8 weeks (August–October 2012) at International Hospital Kampala, an urban Kampala hospital, examining the performance of various health worker cadres after training in the use of a non-surgical device (PrePex). The prospective study obtained approval from the Makerere School of Medicine Research and Ethics Committee and the Uganda National Council of Science and Technology. If eligible, and after the subject signed the informed consent form, they were enrolled into the study.

**Results:**

Ten health workers were successfully trained in use of PrePex during a 3 day non-residential on-the-job training course. After the first three days of training, the trained health workers performed 561 placements and 529 device removals successfully. Over all adverse events (AE) rates were below ≤2%; however, there were some differences in AE rates across the cadres trained but not significant (p>0.25 for moderate AEs).

**Conclusion:**

Rapid training for safe use of the PrePex device is feasible for the range of health workers available for SMC in resource limited settings, but among those with past SMC experience.

## Introduction

In 2012 it was estimated that 35.3 million people were living with HIV and that there were 2.3 million new infections during that year [1]. Discovering ways to prevent the transmission of HIV is of primary concern to health care authorities worldwide.

It is well known from a range of observational and epidemiological studies that the risk of acquiring HIV among heterosexual males can be significantly reduced by 60% through safe male circumcision (SMC) [2], [3]. Numerous papers on the topic have been published over the past two decades to elevate HIV prevention awareness, especially in sub-Saharan countries [2], [3], [4]. A modeling study published in 2009 estimated that scaling up SMC to reach 80% of adult males in 14 African countries by 2015 could potentially avert more than 4 million adult HIV infections between 2009 and 2025 and yield annual cost savings of US$1.4–1.8 billion after 2015, with a total net savings of US$20.2 billion between 2009 and 2025[5].

To date, there are over 38 million adolescent and adult males in Africa who could benefit from SMC for HIV prevention. The challenge Africa faces is how to safely scale up a surgical procedure in resource limited settings. Uganda has a national plan to offer a voluntary SMC program to 4.2 million adult men over 5 years as part of a comprehensive HIV prevention strategy [5]. To achieve this, a minimum of 820,000 procedures need to be performed per year, however over the past 18 months, this target has not been met, falling short by 250,000 procedures. Approaches that involve quicker but equally safe or safer methods are urgently needed to realize scale up and hopefully reach the set targets [5], [6]. In Uganda, one PrePex pilot study has been published and in addition an active surveillance exercise is under way at four sites. In this paper we describe the skills transfer process for a new non- surgical male circumcision device PrePex at a Ugandan SMC site.

## Methods

### Ethical consideration

This study obtained approval from the Makerere School of Medicine Research and Ethics Committee and the Uganda National Council of Science and Technology. If eligible, and after the subject signed the written informed consent form (one-on-one with the principal investigator or designee), he was enrolled into the study.

### Study methods

The training study took place in the context of a prospective study of PrePex safety when used at an urban SMC site, (International Hospital Kampala) conducted from August to October 2012 [7]. A total of 625 subjects were eligible for device placement and removal. Those enrolled were males scheduled to undergo voluntary SMC in an effort to prevent the spread of HIV in resource limited high prevalence settings. Duration of the training period was 3 days and the entire period of the study was eight weeks.

### The training model

An eligible trainee was a SMC certified health worker with prior surgical male circumcision experience of at least 50 SMC cases and working as the primary ‘surgeon’.

Teaching methods included a seminar, ‘dry’ laboratory practice sessions on models and hands-on practice. Materials available for use were: a PrePex video clip [8]. (www.prepex.com/clinical procedure.aspx), pictorial paper charts, PrePex demonstration kits, standardized adverse events (AEs) definitions and Standard Operating Procedures (SoPs) for AE management. Assessment and feedback was conducted in real time through face-to-face sessions between the Prepex master and the trainee. During the assessment of performance, reference was made to the steps (tasks) as indicated in table 1 and 2. Files S1 and S2 show some of the tools used.

**Table 1:** Specific PrePex device placement maneuvers at Kampala IHK site, Uganda 2013.

Maneuvers	Operator	Assistant (required)
*Talks to client (re-assures)	√	√
Re-evaluates prepuce, and glans for suitability of device	√	-
Opens device pack	-	√
Puts on gloves	√	√
*Cleans the penis using antiseptic gauze, then dries	√	√
*Measures penis correct place. Selects correct PrePex size	√	√
Marks the circumcision line correctly (oblique on ventral side-not too sharp at apex)	√	-
Applies 5% lidocaine cream in appropriate amounts	-	√
Places the Placement Ring at base of penis, correct orientation	√	-
Holds open the foreskin wide from both sides with fingers	√	-
Grasps the top of the foreskin after insertion (NA when performed by the Assistant)	√	-
Aligns Elastic Ring with Inner Ring	√	-
Adjusts the foreskin to match the circumcision line	√	-
Releases the Elastic Ring gently, one notch at a time	√	-
Reviews 360 degree around. Checks inner Ring correct position.	√	-
*Removes of verification thread	√	√
Tells the client to get dressed. Discharges client to steward to attend post Placement discharge session	√	√

*Maneuver can be performed by either.

**Table 2:** Specific PrePex device removal maneuvers at Kampala IHK site, Uganda 2013.

Maneuvers (Tasks)	Operator	Assistant (optional)
*Talks to client (re-assures)	√	√
Opens device pack (set)	√	-
Puts on gloves	√	√
Cleans the necrotic foreskin using antiseptic gauze and separates foreskin from glans	√	-
Takes forceps in hand	√	-
Pulls penis upwards, grasps necrotic foreskin with forceps	√	-
Locks forceps before cutting, places them at correct position, transfers them to left hand	√	-
Takes scissors and starts cutting foreskin. Cuts diagonally	√	-
Knows how to change direction while cutting. Cuts efficiently. Cuts close to Inner Ring	√	-
Disposes of foreskin	√	-
Holds scalpel vertically, Inner Ring flat side is facing head of penis. Cuts Elastic Ring on the flat side	√	-
If necessary: places spatula on curved side (not flat side) of Inner Ring to detach foreskin	√	-
Extracts the Inner Ring firmly and quickly	√	-
Reviews the penis	√	-
Cleans the penis with antiseptic solution according to local guidelines	√	-
Checks for oozing or bleeding and applies pressure if necessary	√	√
*Dresses the penis	√	√
Tells the client to get dressed. Discharges client to steward to attend post Removal discharge session	√	√

*Maneuver can be performed by either.

The Training Model contained two parts.

#### Part I– Didactic and Simulation sessions

These were conducted in one large room; with one side lecture room, with chairs, a flip charts, LCP Projector and white board screen. Three lecturers (2 PrePex supervisors and 1 product specialist) conducted the lectures and demonstrations. The following were covered in these sessions: PrePex device, PrePex procedure, tools and materials, male genital anatomy, PrePex screening procedure, PrePex removal procedure, managing client flow, the post procedure healing course, the possible side effects and adverse events. File S3 shows a sample of the training plan used.

Space to accommodate 3 work stations was set up in the other half of the room. One station had a wooden mannequin for the male genitalia, the second station had PrePex device for placement, and the third had PrePex materials for removal [7].

An MCQ examination was conducted at the end of the session. Feedback was given to each participant

#### Part II- Clinical

This was conducted over two days, included screening placement and removal. The time between placement and removal was 6 days (i.e. removal was done on day 7). Ten providers (3 physicians, 2 clinical officers and 5 nurses) were assigned to five training teams. Three PrePex masters trained the teams. A Clinical Officer cadre is equivalent to a Physician Assistant Cadre. Of the 625 eligible men for device placement and removal, 40 were assigned to each team.

### Assessment of Competence

The first 20 procedures for each team were closely monitored and tutored by a PrePex master from Rwanda experienced in performing PrePex procedures. Each trainee was assessed for PrePex knowledge and skills using a predetermined criterion.

Testing of clinical competence which allowed decisions to be made about fitness to perform the procedure (practice) by the trainee, was based on directly observing the individual steps performed correctly. Judgment of whether the steps were correctly performed was a global rating (a point-scale of either needs to improve or competently performed procedure in proper sequence and progressed from the step to step efficiently). Feedback was given instant for either correctly done or not correctly done and requiring a repeat (formative assessment). At the end of the training a certificate was issued to each individual to certify competence. She/he began performing placement and removal of the devices independently. The procedure steps for placement and removal are outlined in tables 1 and 2, respectively and in the supplementary files.

### Data Collection

Rate of adverse events, time taken to perform procedures and number of device placements and removals were collected for each trainee during the eight weeks when devices were placed and removed. A questionnaire was used to collect training outcome data, and these data were analysed for frequencies and trends.

## Results

In total, 625 placements and removals were performed by trainees and trainers. Those trained included five nurses, two clinical officers, three physicians as shown in table 3. All the trainees passed. After the 3 days of training, all trained workers performed procedures during the rest of the study/project period (561 placements and 529 device removals). The majority of procedures were performed by nurses and clinical officers. There was one AE for every 38 placements by a nurse and 1 AE for every 72 placements by a clinical officer and none for surgeons and the trainers. There was one painful (defined as pain score ≥8) removal for every 4 removals by a nurse, 1 in 11 removals by a clinical officer, 1 in 5 by a specialist surgeon and 1 in 2 by the trainers.

**Table 3:** AEs by trainee category at Kampala IHK site, Uganda 2013.

	Nurses (n = 5)	Clinical officer (n = 3)	Medical†† officer (n = 1)	Surgeons†† (n = 2)	Trainer (n = 3)	Total
Placements	309	143	65	44	64	625
Removals	262	169	56	42	6	535
*Moderate AE	8	2	0	0	0	10
†Pain score ≥8 on removal	68 (1∶4)	15 (1∶11)	0	8 (1∶5)	3 (1∶2)	94 (1∶6)
Personnel AE rates	1∶39	1∶72	No AE	No AE	No AE	-
Removal pain rates	1∶4	1∶11	-	1∶5	1∶2	1∶6

*AEs other than pain included bleeding at removal and device displacement due to wrong placement.

† Pain ≥8 on the VAS was graded as a mild AE.

†† Also referred to physicians.

The p- value was >0.25 for differences in moderate AEs between nurses and clinical officers. X^2^ = 0.9 df = 1.

The 10 trainees were enrolled trained and performed procedures over the eight-week enrollment period of the study. There were some differences in AEs among physicians compared with non-physicians in using PrePex during the 8 weeks. All the AEs resolved without sequel.

As shown in table 4, some ‘difficult’ situations were encountered while performing placements; a borderline or narrow prepuce leads to inner ring insertion difficulties. The elastic ring would repeatedly disengage from the inner ring groove and in a separate incident a client had repeated frequent erections, which made device placement impossible.

**Table 4:** Difficult placements and removals.

Event	Number
Borderline (narrow) prepuce	5
Elastic ring disengaging from groove repeatedly	5
Failure to maintain a flaccid penis (repeated erections on table)	1
Difficult removals: Unyielding dried necrotic skin	2

## Discussion

This paper describes a rapid training model for the safe use a of non-surgical circumcision device (PrePex). All 10 trainees were competent in carrying out surgical safe male circumcision. The PrePex screening, placement, removal and counseling skills were mastered with relative ease. AE management was within the capability (competence) of the trainees as they had prior surgical SMC experience.

AE rates occurring when nurses performed the procedures were twice as high as when clinical officers performed the procedures. The physicians posted no AEs, perhaps because the numbers of procedures they performed were less than for the nurses. The moderate AE rates of 1 in every 39 clients (2.6%) is close to or within the generally acceptable AE rate for SMC of 2–5%, suggesting that nurses were safe operators for this device. The p- value was >0.25 for differences in moderate AEs between nurses and clinical officers.

The occurrence of pain during removal on day 7 among some of the participants is undesirable even if it is short lived pain (less than a few seconds). In this study, one in six experienced short lived pain ≥8 (on the VAS) on removal. The trainers had a much higher rate (1 in 2) compared to the rest of the operators; possibly because they performed far fewer removals, they removed only six. So this could be a chance occurrence. Although the reduction or elimination of pain occurrence during device removal may be achieved through practice (experience), there is room to explore other factors that might contribute such as removal, prior counseling, analgesia, etc.

The desirability for short rapid training fits in with a move towards shorter training periods for surgical or procedural skills transfer and emphasis on operating room efficiency. In these trainings, sheer volume of work/procedure exposure rather than specifically designed curricula, is the hallmark of surgical training and skills transfer [9], [10].

The process of new skills training techniques is based on established theories of the ways in which motor skills are acquired and expertise is developed. Fitts and Posner's three-stage theory of motor skill acquisition is widely accepted in the motor skills and surgical literature. In the cognitive stage (the first stage), the learner intellectualizes the task; at this stage performance is erratic, and the procedure is carried out in distinct steps. With practice and feedback, the learner reaches the integrative stage (the second stage) in which knowledge is translated into appropriate motor behaviour. The learner is still thinking about how to move the hands and execute the task with fewer interruptions. In the autonomous stage, (the third stage), practice gradually results in smooth performance. The learner no longer needs to think about how to execute this particular task and can concentrate on other aspects of the procedure [11], [12]. These processes and stages were observed in the 3 days of training and the 7 weeks of observation in this study. We contend therefore that AEs are likely to reduce the more procedures one does.

The duration of time in a profession does not necessarily lead to the development of expertise [13]. It is well reported that development of expertise is dependent on deliberate efforts to change important aspects of performance rather than repetitive execution of routine work [14]. In order to acquire expertise, practice should be challenging in relation to its level of difficulty, informative due to the availability of feedback and repetitive with an opportunity to detect and correct errors [15]. The tasks encountered by our trainees were challenging and of interest in the sense that the device was a new concept, the trainees had no prior experience of the device though the technical concepts underpinning device circumcision are the same as for surgical SMC.

The difficulties pointed out in table 4 highlight some of the challenges encountered. In the context of limited resources, methods for expediting the pathway to expert performance are essential. Anecdotal evidence traditionally attributes development of expertise to experience accumulated. Mere accumulation may not be sufficient for one to become an expert [15], [16], [17], [18]. For some, performance may decrease after training and there are numerous instances where experience and the amount of knowledge and performance are incongruent [19]. Apart from sheer experience and knowledge, other attributes such as behaviour traits, learning styles and environments conducive for the development of expertise should not be lost or left out. Behaviour traits and learning styles were not individually considered in this study though the environment was conducive for learning, was spacious, well-lit, with comfortable ambient temperatures, free of noise, private and non-threatening. Deliberate practice relates to activities in which learners engage with the specific aim of improving some aspect of performance. In this training model, trainers facilitated trainee progression by creating an environment where the trainees could perform repetitive behaviors and receive feedback and instruction to ensure development of key skills.

The steps for placement and removal were clearly stated and it was possible to be instructed upon accordingly. The steps were not complex and repetition was possible. Repeated practice is believed to aid mastery [20]. Assessment was objective, and used a checklist similar to the one used for OSCE (Objective Structured Clinical Examination) [21]. Incorrect placement or removal was apparent before the client would leave the room and therefore there were immediate feedback opportunities. In the context of SMC, PrePex skills transfer training is feasible after a short period of didactic lessons and with on job training for all eligible health workers or practitioners [22]. WHO recently issued guidelines on the use of male circumcision devices. These guidelines derive from studies conducted at several sites in different countries [23], [24].

## Study Limitations

This study only included those operators that had significant prior surgical SMC experience; extrapolation to those without surgical SMC experience may be done with caution. What this study does not explore is when retraining may be required. In the current context there may be time lapses between training in a research setting to routine PrePex practice.

## Conclusions

PrePex device skills are relatively easy to transfer to non-physician and physician cadres in a short duration in resource limited settings, but among those with past SMC experience.

## Supporting Information

File S1 **PrePex Assessment of Trainees Clinical Skills for Screening.**
(PDF)

File S2 **PrePex Assessment of Trainees Clinical Skills for Placement & Removal.**
(PDF)

File S3 **Sample PrePex Training Study Plan for Uganda.**
(DOC)
