# Supplementary material for: Skills Training of Health Workers in the Use of a Non Surgical Device (PrePex) for Adult Safe Male Circumcision
Source: PLoS One. 2014 Aug 13;9(8):e104893. doi: 10.1371/journal.pone.0104893 (PMC4132017; doi:10.1371/journal.pone.0104893)
Supplement: File S1 — PrePex Assessment of Trainees Clinical Skills for Screening. (PDF) [file pone.0104893.s001.pdf]

ASSESSMENT OF TRAINEE'S CLINICAL SKILLS FOR SCREENING

Evaluator / Trainer's Name: ________________________

Date: _____/_____/_____

Trainee is assessed every 3rd client.

Assesses rotate every 2 or 3 clients

Mark each task / activity:

1 = Needs improvement – steps were not performed correctly and/or out of sequence or omitted

2 = Competency performed in proper sequence and progressed from step to step efficiently

3 = Proficiently performed = Steps performed in proper sequence and proficiently performed in proper sequence

N/O = not observed

Trainee's Name: ______________________________ Date: _____/_____/_____

TRAINEE PERFORMANCE OF PREPEX SCREENING PROCEDURE

1 = Needs improvement – steps were not performed correctly and/or out of sequence or omitted
2 = Competency performed in proper sequence and progressed from step to step efficiently
3 = Proficiently performed = Steps performed in proper sequence and proficiently performed in proper sequence
N/O = not observed

Activity	1	2	3
1. Operator introduces himself. Both seated. Asks client's name. Explains purpose of screening.			
2. Interviews client correctly, using part 1 of 'PrePex Screening checklist'			
3. Puts on gloves. Asks client to stand with pants down			
4. Examines client correctly, using part 2 of 'PrePex Screening checklist'			
5. Correctly assesses client's suitability or unsuitability for PrePex MC			

**Knowledge of procedure, procedure flow, time and motion**	1<br>Insufficient knowledge of procedure.<br>Unsure and hesitant.	2<br>Conducts the procedure satisfactorily.	3<br>Confident, flowing, moves easily from one aspect to the next
**OVERALL EVALUATION**	**Not yet competent – NOT recommended for further PrePex training**	**Not yet competent: Recommended for repeat PrePex training course**	**Adequately competent to continue with clinical work under observation of doctor**
