# Supplementary material for: Skills Training of Health Workers in the Use of a Non Surgical Device (PrePex) for Adult Safe Male Circumcision
Source: PLoS One. 2014 Aug 13;9(8):e104893. doi: 10.1371/journal.pone.0104893 (PMC4132017; doi:10.1371/journal.pone.0104893)
Supplement: File S2 — PrePex Assessment of Trainees Clinical Skills for Placement & Removal. (PDF) [file pone.0104893.s002.pdf]

ASSESSMENT OF TRAINEE'S CLINICAL SKILLS FOR PLACEMENT & REMOVAL

Operator Trainee: ____________________________

Assistant Trainee: ____________________________

Trainee is assessed every 3rd client.

Assesses rotate every 2 or 3 clients

Mark each task / activity:

1 = Needs improvement – steps were not performed correctly and/or out of sequence or omitted

2 = Competency performed in proper sequence and progressed from step to step efficiently

3 = Proficiently performed = Steps performed in proper sequence and proficiently performed in proper sequence

N/O = not observed

Evaluator / Trainer's Name: ______________________________ Date: _____/_____/_____

OPERATOR PERFORMANCE OF PREPEX PLACEMENT PROCEDURE

1 = Needs improvement – steps were not performed correctly and/or out of sequence or omitted
2 = Competency performed in proper sequence and progressed from step to step efficiently
3 = Proficiently performed = Steps performed in proper sequence and proficiently performed in proper sequence
N/O = not observed

Task	Client number				
	**1**	**2**	**3**	**4**	**5**
1. Puts on gloves.					
2. Cleans the penis using antiseptic gauze, then dries					
3. Measures penis in correct place. Selects correct PrePex size.					
4. Marks the circumcision line correctly (oblique on ventral side - not too sharp at apex)					
5. Places the Placement Ring at base of penis, correct orientation					
6. Holds open the foreskin wide from both sides with fingers					
7. Grasps the top of the foreskin after insertion (NA when performed by the Assistant)					
8. Aligns Elastic Ring with Inner Ring					
9. Adjusts the foreskin to match the circumcision line					
10. Releases the Elastic Ring gently, one notch at a time					
11. Reviews 360 degree around. Checks Inner Ring correct position.					
12. Tells the client to get dressed. Discharges client to steward to attend post Placement discharge session.					

**Tissue handling. Knowledge of procedure.<br>Device and instrument handling.<br>Procedure flow. Time and motion.**	1<br>Rough or clumsy Awkward and unsure with repeated incorrect moves	2<br>Satisfactory	3<br>Consistently handled tissue appropriately. Good handling of materials.	
**Dealing with unexpected matters**	1<br>Becomes confused and flustered. Does not relate to client appropriately	2<br>Satisfactory	3<br>Remains calm. Keeps client calm, Deals with it appropriately.	N/O

**OVERALL EVALUATION**	**Not yet competent –<br>NOT recommended for further PrePex training**	**Not yet competent:<br>Recommended for<br>repeat PrePex training course**	**Adequately competent<br>to continue with clinical work under observation of doctor**

Evaluator / Trainer's Name: ______________________________ Date: _____/_____/_____

OPERATOR PERFORMANCE OF PREPEX REMOVAL PROCEDURE

1 = Needs improvement – steps were not performed correctly and/or out of sequence or omitted
2 = Competency performed in proper sequence and progressed from step to step efficiently
3 = Proficiently performed = Steps performed in proper sequence and proficiently performed in proper sequence
N/O = not observed

Task	Client number				
	**1**	**2**	**3**	**4**	**5**
1. Puts on gloves					
2. Cleans the necrotic foreskin using antiseptic gauze and separates foreskin from glans					
3. Takes forceps in right hand					
4. Pulls penis upwards, grasps necrotic foreskin with forceps.					
5. Locks forceps before cutting, places them at correct position, transfers them to left hand.					
6. Takes scissors and starts cutting foreskin. Cuts diagonally					
7. Knows how to change direction while cutting. Cuts efficiently. Cuts close to Inner Ring.					
8. Disposes of foreskin					
9. Holds scalpel vertically, Inner Ring flat side is facing head of penis. Cuts Elastic Ring on the flat side.					
10. If necessary: places spatula on curved side (not flat side) of Inner Ring to detach foreskin					
11. Extracts the Inner Ring firmly and quickly					
12. Reviews the penis					
13. Cleans the penis with antiseptic solution according to local guidelines					
14. Checks for oozing or bleeding and applies pressure if necessary					
15. Disinfects the penis with Betadine					
16. Dresses the penis					
17. Tells the client to get dressed. Discharges client to steward to attend post Removal discharge session.					

**Tissue handling. Knowledge of procedure. Good handling, Procedure flow. Time & motion.**	1<br>Rough or clumsy, unsure with repeated incorrect moves	2<br>Satisfactory	3<br>Consistently handled tissue appropriately. Good handling of materials.	
**Dealing with unexpected matters**	1<br>Becomes confused and flustered. Does not relate to client appropriately	2<br>Satisfactory	3<br>Calm. Keeps client calm, Deals with it appropriately.	N/O
**OVERALL EVALUATION**	**Not yet competent – NOT recommended for further PrePex training**	**Not yet competent: Recommended for repeat PrePex training course**	**Adequately competent to continue with clinical work under observation of doctor**	

Evaluator / Trainer's Name: ______________________________ Date: _____/_____/_____

ASSISTANT PERFORMANCE OF PREPEX PLACEMENT PROCEDUR

1 = Needs improvement – steps were not performed correctly and/or out of sequence or omitted
2 = Competency performed in proper sequence and progressed from step to step efficiently
3 = Proficiently performed = Steps performed in proper sequence and proficiently performed in proper sequence
N/O = not observed

Task	Client number				
	**1**	**2**	**3**	**4**	**5**
1. Puts on gloves.					
2. Assists Operator correctly, handing correct materials and tools as needed					
3. Selects correct device size					
4. Applies 1 gr anesthetic cream to whole glans					
5. Inserts the Inner Ring correctly, Flat side on the frenulum					
6. Grasps the top of the foreskin after insertion					
7. Cuts the verification thread					
8. Disposes of materials correctly					

**Tissue handling. Knowledge of procedure.<br>Device and instrument handling.<br>Procedure flow. Time and motion.**	1<br>Rough or clumsy Awkward and unsure with repeated incorrect moves	2<br>Satisfactory	3<br>Consistently handled tissue appropriately. Good handling of materials.	
**Dealing with unexpected matters**	1<br>Becomes confused and flustered. Does not relate to client appropriately	2<br>Satisfactory	3<br>Remains calm. Keeps client calm, Deals with it appropriately.	N/O

**OVERALL EVALUATION**	**Not yet competent –<br>NOT recommended for further PrePex training**	**Not yet competent:<br>Recommended for<br>repeat PrePex training course**	**Adequately competent<br>to continue with clinical work under observation of doctor**

Evaluator / Trainer's Name: _____________________________ Date: _____/_____/_____

ASSISTANT PERFORMANCE OF PREPEX REMOVAL PROCEDURE

1 = Needs improvement – steps were not performed correctly and/or out of sequence or omitted
2 = Competency performed in proper sequence and progressed from step to step efficiently
3 = Proficiently performed = Steps performed in proper sequence and proficiently performed in proper sequence
N/O = not observed

Task	Client number				
	**1**	**2**	**3**	**4**	**5**
1. Puts on gloves					
2. Assists Operator correctly, handing correct materials and tools as needed					
3. Disposes of materials correctly (including cutting Inner Ring)					

**Knowledge of procedure.**<br>**Time and motion.**	1<br>Rough or clumsy Awkward and unsure with repeated incorrect moves	2<br>Satisfactory	3<br>Consistently, correctly handling of materials.	
**Dealing with unexpected matters**	1<br>Becomes confused and flustered. Does not relate to client appropriately.	2<br>Satisfactory	3<br>Remains calm. Keeps client calm, Deals with it appropriately.	N/O

**OVERALL EVALUATION**	**Not yet competent –**<br>**NOT recommended for further PrePex training**	**Not yet competent:**<br>**Recommended for repeat PrePex training course**	**Adequately competent to continue with clinical work under observation of doctor**
