# Supplementary material for: Skills Training of Health Workers in the Use of a Non Surgical Device (PrePex) for Adult Safe Male Circumcision
Source: PLoS One. 2014 Aug 13;9(8):e104893. doi: 10.1371/journal.pone.0104893 (PMC4132017; doi:10.1371/journal.pone.0104893)
Supplement: File S3 — Sample PrePex Training Study Plan for Uganda. (DOC) [file pone.0104893.s003.doc]

**PrePex Training & Study Plan**

**May 24, 2012**

**Training of4teams of physicians and nurses in Ugandato perform safe and effectivePrePex procedures by Rwanda PrePex training center**

**Course Language –** English or French

**Course Duration –** 3 days plus

**Initial Supervision Period (Placements & Removals) –** up to 6 days to supervise at least 30 procedures per team

**Intended trainees –**Physicians &Nurses

**Hours -**8:30 to 15:30

**Special Requirement -**A PrePex Operator will need to come to Uganda a week before training to perform PrePex Placements

**Course Description**

**Objective**

The objectives of the PrePex training course are:

- Learn how to screen suitable clients for PrePex procedure. Identify cases as diseases, abnormalities, special conditions, etc.
- Learn the skills of performing safe and effective PrePexPlacements
- Learn the skills of performing safe and effective PrePexRemovals
- Reassure training efficacy by immediate supervision period.

**Goal**

The PrePextraining course enables establishment of skilled PrePex teams which will be able to perform PrePexMC procedures in a mannerintended for scaling up MC.

**Methodsand Issues to be covered**

- PrePex device
- PrePex procedure tools and materials
- Genital anatomy
- PrePex screening procedure
- PrePex Placement procedure
- PrePex Removal procedure
- Effective PrePex MC flow
- General knowledge of post procedure counseling
- General knowledge of post procedure healing course
- General knowledge of possible side effects and Adverse Events.

**PrePex course outcomes for decision makers:**

A trainee who successfully completes the Operator / Assistant PrePex course will

- Receive a course grade which will aid decision makers to determine his/ her suitability for PrePex
- Be selected as a PrePex Operator or a PrePex Assistant
- Be supervised for ongoing procedures until supervisor certifies his/her effectiveness

**Training Resources Requirements**

- Training HR (supplied by Rwanda):
  1. 1 Training manager
  2. 3PrePexsupervisors
  3. 1 PrePex supervisorfor the immediatesupervision period
  4. 1 PrePex Product Specialist (by manufacturer)
  5. 1 PrePex expert to perform Placements in Uganda, a week before the training initiates (in order to have Removal demonstration subjects in the training)
- Training specialized equipment and documents:
  1. Training presentations:
- Chapter 0 Welcome – course introduction
- Chapter 1 Introduction – PrePex goal
- Chapter 2 Anatomy – basic penis anatomy
- Chapter 3 PrePex device –introducing the PrePex device components
- Chapter 4 Screening - Contraindications for PrePex screening
- Chapter 5 Placement – description of the Placement procedure
- Chapter 6 Removal - description of the Removal procedure
- Chapter 7 - Side effects Complications – indicates how to identify side effects and adverse event
  1. Documents used during training:
     - Training manuals – all printed training presentations (see above details)
     - Training exams – summaries and validates the training course assimilation
     - Training exams answers – correct answers of the written exam
     - How to conduct a written assessment –explains how the exam should be handled
     - Trainee clinical assessment – clinical evaluation form, which summarizes the clinical performance of the trainee
     - Training certificates – granted at the end of the training course which qualifies the trainee to perform PrePex procedures
     - Complete training program schedule – details the course timelines and activities
     - Placement & Removal procedures flow – a sequence of photos describing the correct order of the procedures
     - Screening Clients for PrePex – a step by step screening questionnaire of theclient for the PrePex screening nurse
     - Post Placement / Removal leaflets – explanation for the client on daily care post each procedure visit
     - PrePex Procedure Education for Clients poster – describes the expected procedure course to clients
     - Training tools and materials list.
- Trainees HR
  1. 2 physicians and 6 nurses, of which 4will become PrePex operators and 4PrePex assistants
- Training facility (in hospital), which includes:
  1. Fully equipped training classrooms for 8 trainees (including projector)
  2. Fully equipped **large**PrePexprocedure room with **PrePexstations**for 4teams
  3. **Detailed requirements can be sent on request**
- Training volunteers
  1. Volunteers for Removals hands on - Having PrePexprocedure 1 weekbefore course
  2. Volunteers for Placementstraining
  3. Volunteers for Supervision period
- Equipment and Tools
  1. PrePex and all PrePex tools for theoretical training needs
  2. PrePex and all PrePex tools for practical training
  3. PrePex and all PrePex tools for initial supervision periodprocedures
- Administrative
  1. Food and beverages for training hours
  2. Certifications

**Training Timeline**

- Course should be scheduled 1 month in advance: the official request and invitation has to be availed 30 days before
- Placement procedures are required 1 week pre course byPrePexphysician
- Course duration – 3 days
- Immediate supervisionperiod – additional up to 6 working days in which each team will supervisefor additional 30 Placements and Removals.
